# Educational inequalities in major depressive disorder over the adult life course: a microsimulation

**DOI:** 10.1093/eurpub/ckac129.264

**Published:** 2022-10-25

**Authors:** A Lepe, LA Hoveling, M Boissonneault, JAA de Beer, SA Reijneveld, MLA de Kroon, AC Liefbroer

**Affiliations:** 1 Health Sciences, UMC Groningen, Groningen, Netherlands; 2 Epidemiology, UMC Groningen, Groningen, Netherlands; 3 NIDI, The Hague, Netherlands; 4 Public Health and Primary Care, KU Leuven, Leuven, Belgium; 5 Sociology, Vrije Universiteit Amsterdam, Amsterdam, Netherlands

## Abstract

**Background:**

Educational inequalities in major depressive disorder (MDD) pose a major challenge. Tackling this issue requires evidence on the long-term impact of intervening on modifiable factors, e.g. behavioural and psychosocial factors. Therefore, we aim to simulate the development of educational inequalities in MDD across the life course, and to assess the impact of intervening on the modifiable factors that contribute to these inequalities.

**Methods:**

We used data from the prospective Dutch Lifelines Cohort Study to estimate the required input for a continuous-time microsimulation. The microsimulation allowed us to estimate the development of educational inequalities in MDD with a synthetic cohort of 500,000 individuals followed from ages 18 to 65, and to assess the potential benefit of intervening on quality of social contacts, health literacy, and smoking.

**Results:**

On average, an additional 19.1% of individuals with low education will ever experience MDD between ages 18 and 65 compared to those with high education (32.0% vs 12.9%, respectively). Additionally, individuals with low education generally will develop MDD 0.9 years earlier (35.6 years vs 36.5 years, respectively) and spend 1.2 years more with MDD (6.2 years vs 5 years, respectively), than individuals with high education. Improving the quality of social contacts in individuals with low education would have the largest impact; it would reduce the inequalities in the prevalence, onset, and duration of MDD by an average of 18.4%, 18.3%, and 28.6%, respectively.

**Conclusions:**

Intervening on modifiable factors, especially quality of social contacts, in individuals with low education could help reduce the large estimated educational inequalities in MDD over the life course.

**Key messages:**

• There are large educational inequalities in major depressive disorder (MDD) over the life course, especially with regard to the life course prevalence of MDD.

• Improving quality of social contacts, and to a lesser extent health literacy and smoking behaviours, amongst individuals with low education may help reduce the inequalities in MDD.

